# Effects of the selective serotonin reuptake inhibitors citalopram and escitalopram on glucolipid metabolism: a systematic review

**DOI:** 10.3389/fendo.2025.1578326

**Published:** 2025-06-17

**Authors:** Yajing Dai, Mingzhe Zhao, Mian Li, JinQi Ding, Mengfei Ye, Zhonglin Tan, Sugai Liang

**Affiliations:** ^1^ The Fourth Clinical Medical College of Zhejiang Chinese Medical University, Hangzhou, China; ^2^ Affiliated Mental Health Centre & Hangzhou Seventh People’s Hospital, Zhejiang University School of Medicine, Hangzhou, China; ^3^ Department of Psychiatry, Shaoxing Seventh People’s Hospital, Shaoxing, Zhejiang, China

**Keywords:** major depressive disorder, type 2 diabetes mellitus, glucolipid metabolism, citalopram, escitalopram, systematic review

## Abstract

**Objectives:**

Type 2 diabetes mellitus (T2DM) and major depressive disorder (MDD) frequently co-occur, highlighting the need to understand the metabolic effects of antidepressants. This systematic review evaluated the impact of citalopram and escitalopram on glucose and lipid metabolism, focusing on glycemic control.

**Methods:**

A comprehensive search of PubMed, Embase, Web of Science, PsycINFO, the Cochrane Library and Google Scholar was conducted. Primary outcomes included changes in glycosylated hemoglobin (HbA1c) and fasting blood glucose (FBG). Secondary outcomes assessed lipid profiles (triglycerides, cholesterol, high-density lipoprotein, and low-density lipoprotein) and depressive symptom scales. Subgroup analyses were conducted to evaluate outcomes in patients with comorbid T2DM and MDD and those with MDD only.

**Results:**

Thirteen studies involving 502 participants met the inclusion criteria. Six randomized controlled trials, four prospective studies, one cohort trial, one single-arm trial and one three-arm trial. The findings suggest that both citalopram and escitalopram tend to reduce HbA1c and FBG levels. No significant effects on lipid profiles were observed across the included studies.

**Conclusion:**

Citalopram and escitalopram appear to exert beneficial effects on glycemic control, as evidenced by reductions in HbA1c and FBG. Further high-quality investigations are warranted to validate these findings and guide individualized treatment strategies.

**Systematic review registration:**

https://www.crd.york.ac.uk/PROSPERO/view/CRD42024544963, identifier CRD42024544963.

## 1 Introduction

The global prevalence of type 2 diabetes mellitus (T2DM) and major depressive disorder (MDD) has increased substantially, posing a significant public health concern (1). In individuals with T2DM, the prevalence of depression is approximately twice that observed in those without T2DM, with women exhibiting a higher rate than men, irrespective of diabetes status (2). This relationship between T2DM and MDD is bidirectional, influenced by a complex interplay of biological, psychological, and social factors (3). These factors worsen depressive symptoms and impair glycemic control, thereby complicating the management of both conditions.

Insulin, a peptide hormone produced by pancreatic beta cells, crosses the blood-brain barrier and binds to endothelial cell receptors, initiating tyrosine kinases-dependent signaling cascades (4). These cascades regulate central and peripheral metabolic processes, including synaptic plasticity, neurotransmitter modulation, and neuro cognitive functions (5, 6). Insulin resistance, an early hallmark of T2DM, disrupts glucose metabolism in muscle, adipose, and hepatic tissues and impairs dopaminergic (DA) signaling and reward-related behaviors, highlighting a critical link between T2DM and MDD (7, 8). Improving central insulin signaling can peripheral insulin sensitivity, reduce glucose production, and optimize metabolic outcomes (9). Chronic stress further aggravates insulin resistance and depressive symptoms through dysregulation of the hypothalamic-pituitary-adrenal (HPA) axis and excessive innate immune activation (10). Oxidative stress augments these pathologies by damaging lipids and proteins and by reducing antioxidant enzyme activity in the brain and pancreas (11).

Selective serotonin reuptake inhibitors (SSRIs) have been shown to improve blood glucose levels compared to placebo, with Fluoxetine and escitalopram/citalopram demonstrating particular efficacy (12). Escitalopram, the S-enantiomer of citalopram, exhibits superior tolerability and safety compared to other antidepressants (13, 14). It may also enhance glycemic control, by modulating the HPA axis (15), increasing insulin sensitivity in hepatic and muscular tissues, and normalizing neural connectivity in regions such as the hippocampus, and nucleus accumbens (NAc) (16–18). Moreover, escitalopram has been reported to exert antioxidant and anti-inflammatory effects and to reduce lipid levels (19–21).

These findings suggest that escitalopram may confer therapeutic benefits for glycemic control in patients with comorbid T2DM and MDD. The present study aims to systematically review current evidence on the effects of citalopram and escitalopram on glucose and lipid metabolism in this population.

## 2 Methods

### 2.1 Research registration

The study protocol was pre-registered with PROSPERO (CRD42024544963) prior to initiating the literature review.

### 2.2 Data source and search methodology

**Figure 1:**
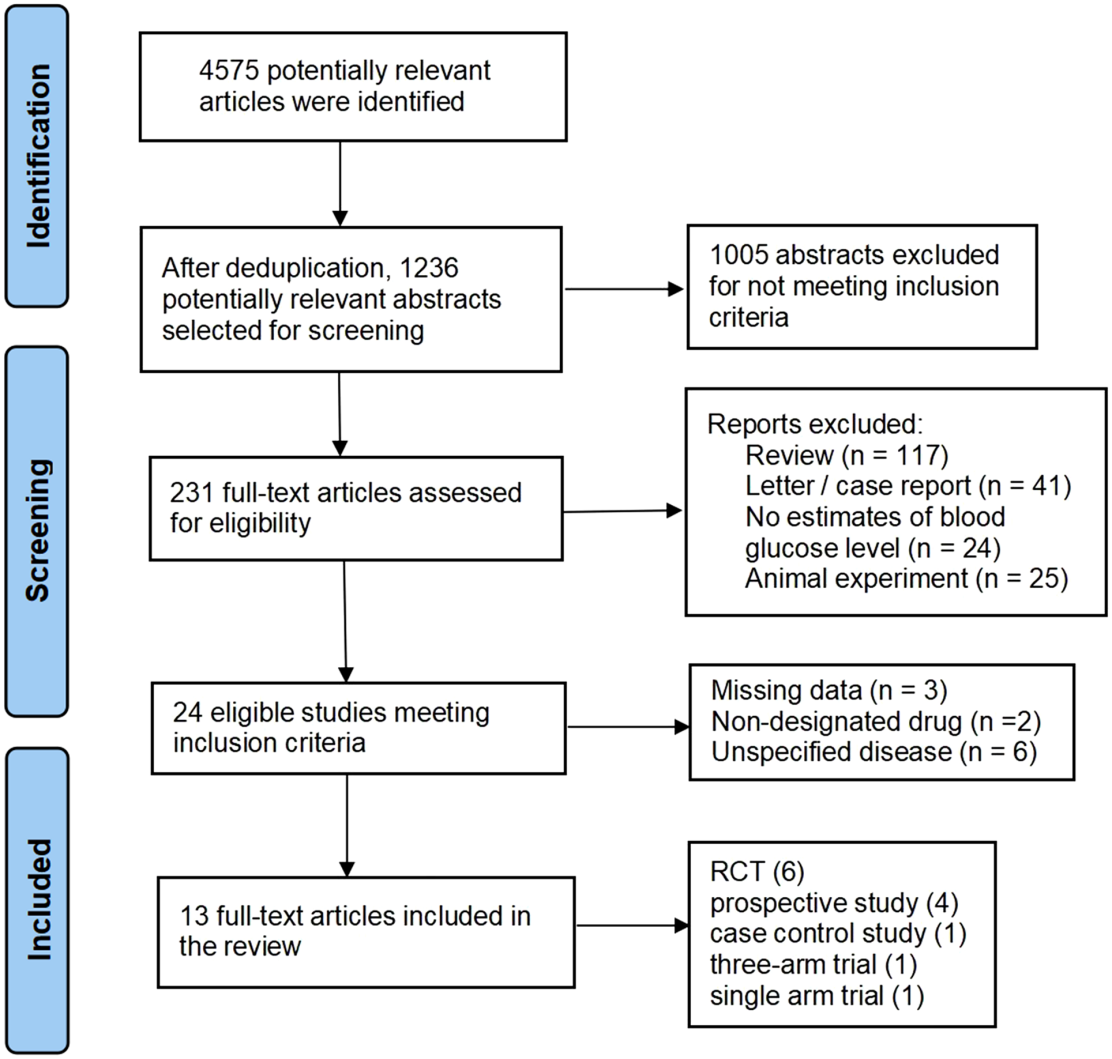
Flowchart of study identification and selection.

A systematic literature review was conducted utilizing databases including PubMed, Embase, Web of Science, Google Scholar, PsycINFO and Cochrane databases. The search, limited to English-language publications and focused on clinical trials investigating the effects of citalopram or escitalopram on glucose metabolism in patients with T2DM from January 2000 to December 2024. Boolean search terms included: (“escitalopram” OR “escitalopram oxalate” OR “citalopram hydrobromide”) AND (“diabetes mellitus, type 2” OR “diabetes” OR “type 2 diabetes” OR “diabetes” OR “serum glucose” OR “glucose metabolism” OR “hyperglycemia” OR “hypoglycemia” OR “glucose metabolism disorder” OR “glycosylated hemoglobin” OR “glucose intolerance” OR “insulin resistance” OR “impaired glucose metabolism”). Title and abstract screening were conducted independently by two reviewers (YJD and ML). Relevant studies were further assessed as detailed in **Figure 1**. Any discrepancies were resolved through discussion, with mediation by SGL when necessary.

### 2.3 Inclusion and exclusion criteria

Inclusion criteria: (1) clinical trials assessing the impact of citalopram or escitalopram on glucose metabolism; (2) studies involving T2DM patients with clinical glucose-related outcomes; (3) English-language publications; and (4) research published between January 2000 and December 2024.

Exclusion criteria: (1) reviews, letters, case reports, cross-sectional studies, and other non-original literature; (2) trials lacking adequate statistical data; (3) studies involving T2DM patients with comorbid severe mental disorders other than MDD; and (4) research on type 1 diabetes, gestational diabetes, or non-T2DM conditions.

### 2.4 Data extraction

Extracted data included authorship, publication year, geographic region, participant demographics (sex, age), study design, sample size, and follow-up duration. Primary outcomes were changes in glycosylated hemoglobin (HbA1c, %) and fasting blood glucose (FBG, mg/dl). Secondary outcomes included lipid profiles including triglycerides (TG, mg/dl), cholesterol (CH, mg/dl), high-density lipoprotein (HDL, mg/dl), and low-density lipoprotein (LDL, mg/dl), and assessments of depressive symptoms.

### 2.5 Quality assessment

The methodological quality of included studies was assessed using the Newcastle-Ottawa Scale, which evaluates three key domains: selection, comparability, and outcome/exposure assessment. Surveys with overall scores of 0–3, 4–6, and 7–9 were categorized as being of poor, fair, or good quality, in that order. We used the Grading of Recommendations, Assessment, Development, and Evaluations (GRADE) to assess the evidence quality (22).

### 2.6 Statistical analysis

Data were summarized as means with standard deviations, where applicable. Study heterogeneity was evaluated using Higgins’ I² statistic and *p* value. The I² statistic was interpreted as follows: no heterogeneity was defined as <25%, mild heterogeneity as 25–50%, moderate heterogeneity as 50–75%, and high heterogeneity as >75% (23). A fixed-effects model was used for low heterogeneity (*p* > 0.1, I² ≤ 50%), and a random-effects model for high heterogeneity (*p* < 0.1, I² > 50%). Funnel plots were generated to inspect plot asymmetry visually. Begg’s and Egger’s regression tests were used.

Sensitivity analyses were conducted to evaluate the robustness of the results. Subgroup analyses were stratified by disease subgroup (T2DM-MDD vs MDD-only) and pharmacological classification (escitalopram vs citalopram), with additional stratification by geographic region and age strata. All statistical analyses were performed using Stata 17.0.

## 3 Results

### 3.1 Characteristics of included studies

The study selection process is shown in **Figure 1**. Of the 231 screened articles, 13 studies met the inclusion criteria, six randomized controlled trials, four prospective studies, one cohort study, one single-arm trial, and one three-arm trial. Quality assessments are illustrated in **Supplementary Figure S1**; **Supplementary Table S1**. The GRADE assessments are listed in **Supplementary Table S2**. Collectively, these studies involved 502 middle-aged and older adults, with 23 participants loss to follow-up. Participants were stratified into two groups: those with comorbid T2DM-MDD and those with MDD only. Two studies assessed citalopram (20–40 mg/day for 8–26 weeks), while nine evaluated escitalopram (5–30 mg/day for 1–52 weeks). Further categorization was based on study region (Middle East, South Asia, East Asia, Western Europe, and North America) and age (<50 years, 50-60 years, >60 years). See **Table 1**.

**Table 1:** Summary of baseline characteristics.

Study	Baseline status	Intervention	Study regions	Age (years)	BMI (kg/m^2^)	Total participants	Main outcomes	Endpoint (weeks)
Khazaie et al. (24)	T2DM+MDD	C:40mg/d	Iran	47.70±8.63	30.64±5.85	20	HbA1c, FBG, BDI	12
Gehlawat et al. (25)	T2DM+MDD	E:10-20mg/d	India	50.75±8.88	26.83±4.41	43	FBG, HbA1c, HAMD, lipid profile	12
Nicolau et al. (26)	T2DM+MDD	C:20mg/d	Spain	60.35±10.73	31.09±4.95	38	HbA1c, BDI	26
Kumar et al. (27)	T2DM+MDD	E:20mg/d	India	48.65±10.19	28.88±4.58	20	FBG, HbA1c, HAMD, HAMA	8
Sebedi et al. (28)	T2DM+MDD	E:10mg/d	Nepal	NR	NR	37	FBG, PPBG	6
Khassawneh et al. (29)	T2DM+MDD	E:5-10mg/d	Jordan	47.68±8.39	31.75±5.81	12	HbA1c, lipid profile, PHQ-9	12
Israt et al. (30)	T2DM+MDD	E:NR	Dhaka	51.2±6.3	NR	35	FBG, PPBG, HbA1c	12
Wei et al. (31)	T2DM+MDD	E:10mg/d	China	43.31±4.57	23.33±1.24	30	FBG, SDS, SAS	1
Santi et al. (32)^1^	T2DM+MDD	E:10-20mg/d	India	42.00±15.93	26.90±1.11	10	FBG, HbA1c, CH, TG, HAMD	12
Shubha et al. (33)	T2DM+MDD	E:5mg/d	India	42.0±3.36	NR	32	HbA1c, MADRS	52
Tiwary et al. (34)	T2DM+MDD	E:NR	India	63.2±10.6	NR	125	FBG, PPBG, HbA1c, HAMD	12
Papakostas et al. (35)	MDD	E:10-30mg/d	Boston	NR	NR	68	FBG, HbA1c, lipid profile	8
Kudyar et al. (36)	MDD	E:10mg/d	India	39.74±8.60	NR	26	FBG, HAMD	6
Santi et al. (32)^2^	MDD	E:10-20mg/d	India	42.00±15.93	26.90±1.11	6	FBG, HbA1c, CH, TG HAMD	12

T2DM, type 2 diabetes. MDD, major depressive disorder. C, citalopram. E, escitalopram. BMI, body mass index. NR, not reported.

HbA1c, glycosylated hemoglobin. FBG, fasting blood glucose. PPBG, postprandial blood glucose. CH, cholesterol. TG, triglyceride. lipid profile includes triglyceride, total cholesterol, high density lipoprotein and lower density lipoprotein.

BDI, Beck Depression Inventory. HAMD, Hamilton Depression Scale. HAMA, Hamilton Anxiety Scale. PHQ-9, Patient Health Questionnaire-9. SDS, Self-Rating Depression Scale. SAS, Self-Rating Anxiety Scale. MADRS, Montgomery-Asberg Depression Rating Scale.

Santi et al. (32) ^1^ represents T2DM comorbid with MDD group, and Santi et al. (32) ^2^ represents MDD only group.

**Figure 2:**
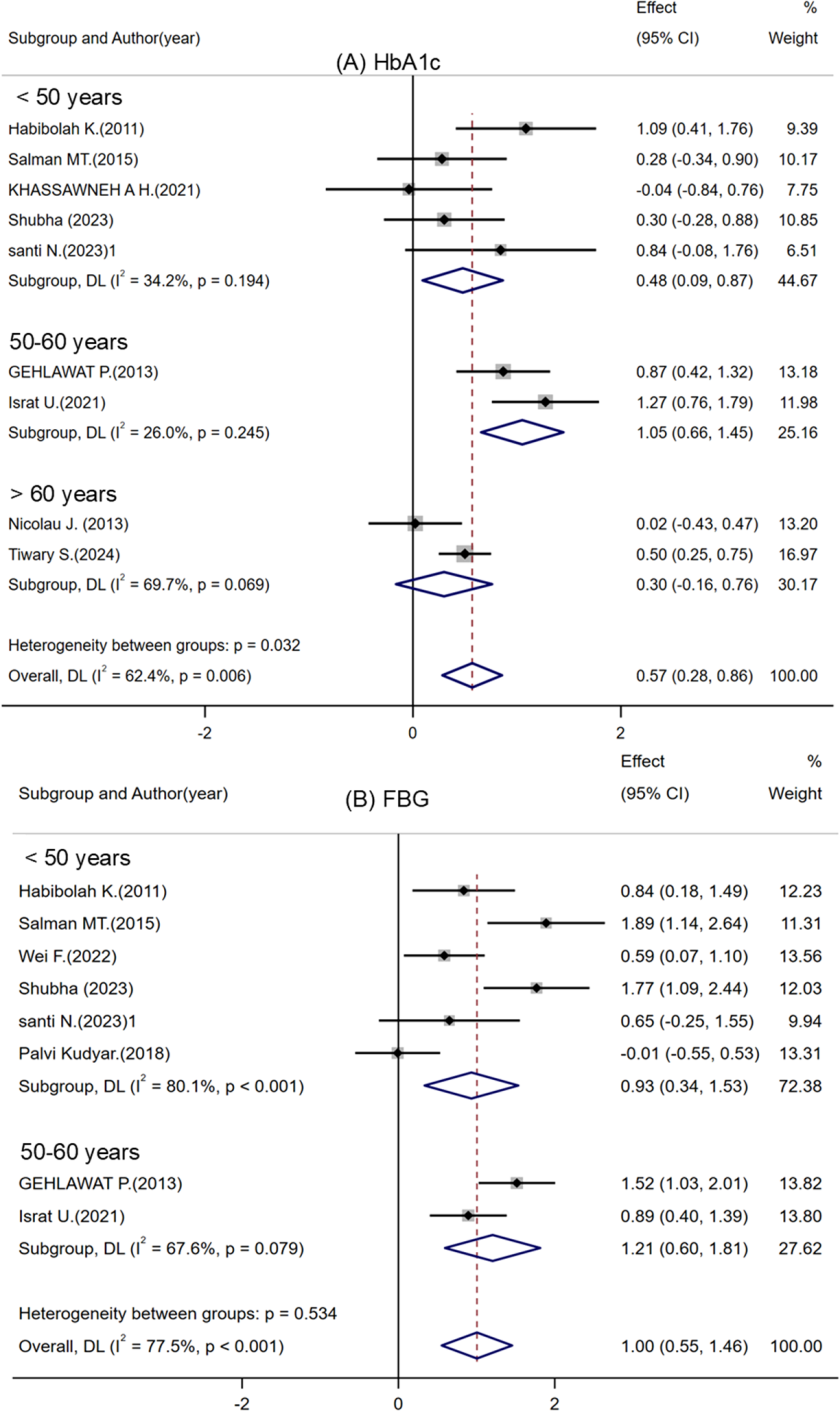
Subgroup analysis of study regions. **(A)** HbA1c (glycosylated hemoglobin) levels before and after treatment. **(B)** FBG (fasting blood glucose) levels before and after treatment. Notes: Santi N. (32)^1^ represents T2DM comorbid with MDD group, and Santi N. (32)^2^ represents MDD only group.

**Figure 3:**
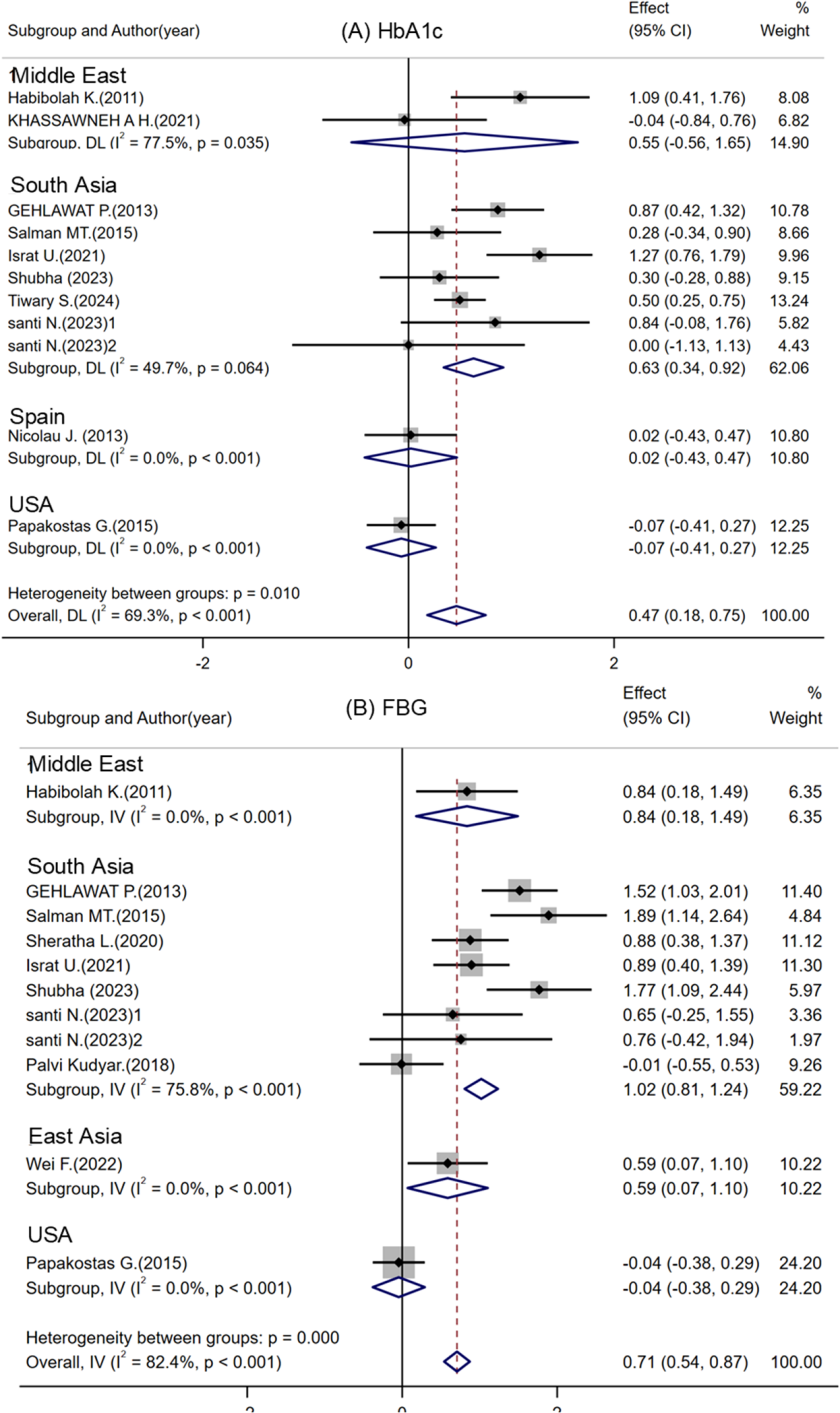
Subgroup analysis of participant age. **(A)** HbA1c levels before and after treatment. **(B)** FBG levels before and after treatment. Notes: Santi N. (32)^1^ represents T2DM comorbid with MDD group, and Santi N. (32)^2^ represents MDD only group.

Due to differences in study designs and outcomes, a formal meta-analysis was not feasible, prompting a systematic review instead. Given substantial between-study heterogeneity (I² > 50%), the results were restricted to outcomes demonstrating low-to-moderate heterogeneity (I² **≤** 50%), with exploratory pooled estimates derived from random-effects models (**Figures 2**, **3**; **S2**). The results of sensitivity analyses are listed in **Supplementary Table S3**. Publication bias was systematically evaluated through funnel plot asymmetry assessments using Begg’s rank correlation and Egger’s weighted regression tests, with graphical representations in **Supplementary Figures S3**, **S4** and quantitative results in **Supplementary Table S4**.

### 3.2 Primary outcome measures

#### 3.2.1 HbA1c

HbA1c, a crucial marker of glycemic control was assessed in ten studies involving 409 participants, with treatment durations ranging from 8 weeks to 12 months (24–27, 29–35). Significant reductions in HbA1c levels were observed in specific subgroups, notably in a South African cohort (standardized mean difference [SMD] = 0.63, 95% CI: 0.34-0.92) (**Figure 2A**). Among participants under 60 years, the SMD was 0.48 (95% CI: 0.09-0.87) for those under 50 years and 1.05 (95% CI: 0.66-1.45) for the 50-60 years group (**Figure 3A**). The forest plot indicates that both citalopram and escitalopram may lower HbA1c levels (**Supplementary Figure S5A**), as supported by disease-specific (**Supplementary Figure S6A**) and drug subgroup analyses (**Supplementary Figure S7A**). High heterogeneity (I² > 50%) likely arise from differences in sample characteristics and study designs.

Four studies reported statistically significant HbA1c reductions. Khazaie et al. (24) documented a 1.59% ± 1.03 decrease (P < 0.001), while Gehlawat et al. (25)Tiwary et al. (34) and Israt et al. (30) also reported significant improvements. Although six studies (26, 27, 29, 31–33, 35) found no significant HbA1c changes, a trend toward improvement was noted.

#### 3.2.2 FBG

FBG was evaluated a primary marker of glycemic fluctuation, was evaluated in ten studies involving 327 participants (24, 25, 27, 28, 28, 30–33, 35, 36). These studies compared the effects of escitalopram or citalopram versus placebo on FBG, over treatment durations of 1 to 12 weeks. Eight studies specifically targeting patients with T2DM-MDD (24, 25, 27, 28, 30–33) reported significant post-treatment improvements in FBG (**Supplementary Figure S5B**). In contrast, no significant FBG changes were noted in patients with MDD only, suggesting that the T2DM-MDD comorbidity uniquely influences glucose metabolism. The forest plot (**Supplementary Figure S6B**) suggests that these medications may reduce FBG levels, supported by study region, age and drug subgroup analyses (**Figures 2B**, **3B**, and **S7B**). High heterogeneity (I² > 50%) may be attributed to variations in sample characteristics and study designs.

Khazaie et al. (24) observed a significant FBG reduction (39.95 ± 25.66 mg/dL, P < 0.001) in T2DM-MDD patients treated with citalopram (40 mg/d). Similarly, Israt et al. (30) found that 12 weeks of escitalopram significantly improved FBG levels in patients with T2DM-MDD (P < 0.001). Additional studies (Wei et al. (31) [P = 0.027] Gehlawat et al. (25) [P < 0.05] Sebedi et al. (28) [P < 0.001]) also reported statistically significant effects, whereas Santi et al. (32) did not observe significant changes (P > 0.05).

### 3.3 Second outcome measures

#### 3.3.1 Lipid profile

Four studies. (25, 29, 32, 35) assessed TG and CH levels before and after escitalopram treatment, finding no statistically significant changes (**Supplementary Table S5**; **Supplementary Figure S8**). Disease subgroup analyses yielded similar results (**Supplementary Figure S9**). Additionally, three studies (25, 29, 35) examined the effects of escitalopram on HDL and LDL levels, reporting no significant differences between escitalopram and placebo. These findings suggest that escitalopram exerts a negligible impact on lipid profiles, including TG, CH, HDL, or LDL level.

#### 3.3.2 Clinical depression assessment

Nine studies assessed changes in depressive symptoms using instruments including the Hamilton Depression Rating Scale (HAMD), the Beck Depression Inventory (BDI), and other relevant depression scales before and after treatment. Although overall interventions alleviated depressive symptoms, statistically significant was not reached across all studies (**Supplementary Figure S10A**). Nonetheless, four studies (25, 32, 34, 36) reported significant reductions in HAMD total scores (**Supplementary Figure S10B**), while two studies (24, 26) observed significant decreases in BDI scores (**Supplementary Figure S10C**), indicating a favorable therapeutic treatment.

## 4 Discussion

This systematic review represents the first comprehensive analysis of the effects of citalopram and escitalopram on glucolipid metabolism. Synthesized data from thirteen studies—primarily involving adults with T2DM-MDD and individuals with MDD only. Our findings suggest that both citalopram and escitalopram tend to reduce HbA1c and FBG levels. However, no significant effects on lipid profiles were observed across the included studies.

These results are consistent with previous reports (25, 30), which demonstrated significant improvements in glycemic control, particularly reductions in FBG and HbA1c, among patients with T2DM and comorbid MDD. The underlying hypothesis guiding this review posits that poor glycemic control in patients with comorbid T2DM and MDD is linked to insulin resistance, which contributes to MDD pathophysiology. A GWAS utilizing data from the UK Biobank identified 496 shared risk SNPs, implicating critical biological pathways involved in both disorders, including glycolipid metabolism (*PPAP2B, DGKB, LIPC*), adipocytokine signaling (*LEPR, PPARGC1A*), T2DM (*GCK, CACNA1C*), long-term depression (*ITPR2, IGF1*), and immune pathways (*NFATC3, NFATC2*) (37). Rodent models of insulin resistance exhibit impaired dopaminergic signaling and disrupted reward-related behaviors (7, 38), reflecting detrimental effects on emotional well-being. A hyperdopaminergic state in the amygdala may underlie increased risk of mood disorder observed in insulin-resistant, diabetic rats (39). Moreover, the high expression of insulin receptor in the dopaminergic neurons of midbrain, which encodes reward-seeking behavior, underscores the interplay between insulin signaling and mood regulation (29, 40, 41). In diabetic patients, peripheral insulin administration markedly impairs glucose metabolism in appetite- and reward-related regions, such as the mesostriatal system, compared to healthy controls (42).

The genotyping of the *CYP2C19* gene is essential for personalizing escitalopram therapy, as the metabolizer status significantly influences drug concentrations and therapeutic efficacy (43). Escitalopram response is intricately to reward processing, with early increases in frontostriatal connectivity during reward anticipation correlating significantly with reduced depressive symptoms after eight weeks of treatment (16). Moreover, in patients with MDD, improvements in depressive symptoms after two weeks of escitalopram treatment were positively correlated with increased functional connectivity between the left hippocampus and the inferior frontal gyrus, suggesting an early predictor of antidepressant efficacy (18). These findings imply that escitalopram may ameliorate both cognitive and emotional functions that are compromised by diabetes. Notably, escitalopram is reported to enhance synaptic plasticity within three to five weeks in healthy individuals (44), positioning it as a promising candidate for improving insulin sensitivity while and simultaneously addressing the dual challenges posed by T2DM and MDD.

Psychological factors, such as stress and depression, also play a significant role in glucose regulation, suggesting a complex interplay between mental health and metabolic processes (45). From an endocrinological perspective, it is essential to explore how antidepressants might affect insulin sensitivity and glucose metabolism. The relationship between T2DM and MDD is primarily mediated through the HPA axis, which drives elevated cortisol levels that adversely affect brain regions rich in glucocorticoid receptors (46–48). Escitalopram has been demonstrated to enhance cognitive function in stressed rodent models by modulating the HPA axis and the insulin receptor substrate/Glycogen Synthase Kinase 3 (GSK-3β) signaling pathway (46), suggesting that escitalopram may represent a viable therapeutic strategy for the concurrent management of T2DM and MDD. Insulin resistance and hyperglycemia contribute to mitochondrial dysfunction, generating reactive oxygen species (ROS) that disrupt energy metabolism and initiate apoptosis (49). In models of chronic stress-induced depression, escitalopram has demonstrated efficacy in mitigating oxidative damage, enhancing antioxidant defenses, and modulating brain-derived neurotrophic factor (BDNF) levels, thereby promoting neuronal healthy (19). Moreover, n-3 polyunsaturated fatty acids (PUFAs) and escitalopram may work synergistically enhance adenylyl cyclase activity and BDNF expression, further reinforcing their antidepressant effects (50). In summary, antidepressants such as escitalopram and citalopram may affect glucose metabolism by addressing stress and depression, which are recognized factors affecting insulin sensitivity and glucose regulation.

Escitalopram therapy significantly reduced CH, TG, LDL and malondialdehyde levels, while increasing HDL compared to the atherosclerosis model group (21). In contrast, antidepressants such as citalopram and escitalopram have been associated with adverse alterations in lipid profiles, including elevated triglyceride levels, increased LDL cholesterol, and decreased HDL cholesterol (51). A 24-month observational study revealed that the use of antidepressants such as escitalopram, paroxetine, and duloxetine was associated with a 10-15% increased risk of weight gain of at least 5% from baseline weight (52). Additionally, atypical depression has been correlated with heightened insulin resistance, characterized by increased appetite and subsequent weight gain (53).

Our results demonstrated no significant alterations in lipid homeostasis, contrasts with prior observational studies reporting modest elevations in TG and CH among individuals T2DM-MDD (29). This discrepancy may be attributed to several factors: (i) population-specific pathophysiological characteristics, (ii) limited longitudinal assessment windows, and (iii) differences in baseline glycemic control. Moreover, obesity-related neuroinflammation has been shown to impair serotonin transporter (SERT) expression in the hippocampus, potentially elucidating the diminished responsiveness to SSRIs observed in obese individuals with comorbid depression (54). Understanding the complex interactions between mental health and lipid metabolism is crucial for developing comprehensive treatment strategies, particularly in patients presenting with comorbid obesity and depression.

Given the pathophysiological insights into depression, the exploration of novel therapeutic strategies is paramount importance. Chronic unpredictable mild stress induces been shown to induce depressive-like behaviors and neuroinflammation in leptin-deficient mice, effects that were reversed by pioglitazone, a peroxisome proliferator-activated receptor gamma (PPARγ) agonist, likely through the enhancement of plasma glucose levels (55). Pioglitazone has emerged as a promising adjunctive treatment for non-diabetic MDD, demonstrating early improvements and potential remission (56). Clinical investigations further substantiate its efficacy and safety, positioning pioglitazone as an augmentation strategy for patients with moderate to severe MDD (57). Moreover, the 5-HT3 receptor antagonist 3-methoxy-N-p-tolylquinoxalin-2-carboxamide (QCM-4) has exhibited considerable promise in improving insulin sensitivity and mitigating depressive-like behaviors in mice subjected to a high-fat diet, while also normalizing glucose and lipid profiles (58, 59).

This study has several limitations. First, significant variability exists among the included studies due to differences in baseline metabolic profiles, medication dosages, and statistical methods. Second, the relatively small sample sizes underscore the need for larger clinical trials to confirm these findings. Third, the exclusion of non-English publications may introduce publication biases. Moreover, individual responses to SSRIs vary, with some patients experiencing dyslipidemia and weight gain as side effects.

## 5 Conclusion

Despite methodological and sample size limitations precluding a formal meta-analysis, the available evidence suggests that both citalopram and escitalopram are effective in reducing FBG and HbA1c levels. To strengthen the evidence base, future research should prioritize large-scale, multicenter RCTs utilizing standardized protocols for treatment duration and dose titration. These studies should include diverse patient populations, stratified by obesity status, diabetes severity, and depression subtypes, to identify subgroups most likely to benefit. Such efforts are essential for validating the efficacy of these medications and for drawing more definitive conclusions regarding their effects on glycemic control.

## Data Availability

The original contributions presented in the study are included in the article/**Supplementary Material**. Further inquiries can be directed to the corresponding authors.
