# Supplementary material for: Effects of the selective serotonin reuptake inhibitors citalopram and escitalopram on glucolipid metabolism: a systematic review
Source: Front Endocrinol (Lausanne). 2025 Jun 17;16:1578326. doi: 10.3389/fendo.2025.1578326 (PMC12209303; doi:10.3389/fendo.2025.1578326)
Supplement: Supplementary file 1 [file DataSheet1.docx]

**Supplementary Materials for *Effects of the selective serotonin reuptake inhibitors citalopram and escitalopram on glucolipid metabolism: a systematic review***

**Supplementary Figures:**

Fig. S1 Risk of bias summary

Fig. S2 Galbraith plot for assessing heterogeneity (Glycemic Profiles)

Fig. S3 Funnel plot for assessing publication bias (Glycemic Profiles)

Fig. S4 Funnel plot for assessing publication bias (Lipid Profiles)

Fig. S5 Changes in glucose metabolism before and after treatment

Fig. S6 Subgroup analysis of T2DM+MDD group and MDD only group

Fig. S7 Subgroup analysis of Escitalopram group and Citalopram group

Fig. S8 Changes in lipid metabolism before and after treatment with Citalopram or Escitalopram

Fig. S9 Subgroup analysis of lipid metabolism changes in T2DM+MDD and MDD only groups

Fig. S10 Changes in depression and anxiety assessment scales before and after treatment with Citalopram or Escitalopram

**Supplementary Tables:**

Table S1 Newcastle-Ottawa Scale of the included studies

Table S2 GRADE summary of evidence

Table S3 Heterogeneity metrics

Table S4 Publication bias assessment

Table S5 Changes in lipid measures of the included studies

**Fig. S1 Risk of bias summary**

| Studies | Selection | | | | Comparability | Outcome | | | Quality Scores |
| --- | --- | --- | --- | --- | --- | --- | --- | --- | --- |
|  | Representativeness of the exposed group | Selection of the non-exposed group | Ascertainment of exposure factors | Demonstration that outcome of interest was non-present at start of study | Comparability of groups on the basis of the design or analysis ^a^ | Assessment of outcome | Was follow-up long enough for outcomes to occur | Adequacy of follow up of groups |  |
| Khazaie et al. (2011) | **☆** | **☆** | **☆** | **☆** | **☆** | **☆** | **☆** | **☆** | 8 |
| Gehlawat et al. (2013) | **☆** | **-** | **☆** | **-** | **☆** | **☆** | **☆** | **☆** | 6 |
| Nicolau et al. (2013) | **☆** | **☆** | **☆** | **-** | **☆** | **☆** | **☆** | **☆** | 7 |
| Papakostas et al. (2015) | **☆** | **☆** | **☆** | **☆** | **☆** | **☆** | **☆** | **☆** | 8 |
| Kumar et al. (2015) | **☆** | **☆** | **☆** | **☆** | **☆** | **☆** | **-** | **☆** | 7 |
| Kudyar et al. (2018) | **☆** | **☆** | **☆** | **☆** | **☆** | **☆** | **-** | **☆** | 7 |
| Subedi et al. (2020) | **☆** | **-** | **☆** | **☆** | **☆** | **☆** | **-** | **-** | 5 |
| Khassawneh et al. (2021) | **☆** | **☆** | **☆** | **-** | **☆** | **☆** | **☆** | **☆** | 7 |
| Wei et al. (2022) | **☆** | **☆** | **☆** | **☆** | **☆** | **☆** | **-** | **☆** | 7 |
| Israt et al. (2022) | **☆** | **☆** | **☆** | **-** | **☆** | **☆** | **-** | **☆** | 7 |
| Shubha et al. (2023) | **☆** | **☆** | **☆** | **☆** | **☆** | **☆** | **☆** | **-** | 7 |
| Santi et al. (2023) | **☆** | **☆** | **☆** | **-** | **☆** | **☆** | **☆** | **☆** | 7 |
| Tiwary et al. (2024) | **☆** | **☆** | **☆** | **-** | **☆** | **☆** | **-** | **☆** | 6 |

^a^ A maximum of two stars can be allotted in this category, one for age, the other for other controlled factors.

**Table S1 Newcastle-Ottawa scale of the included studies**

**Table S2 GRADE summary of evidence**

| Outcomes | Certainty assessment | | | | | Effect | | Total subjects | Certainty of the evidence |
| --- | --- | --- | --- | --- | --- | --- | --- | --- | --- |
|  | Risk of Bias | Inconsistency | Indirectness | Imprecision | Publication Bias | Relative  (SMD [95%CI]) | Absolute (95%CI) |  |  |
| FBG level | not serious | serious | not serious | not serious | not serious | 0.71 (0.54,0.87) | - | 327 | ⨁⨁⨁◯ Moderate |
| HbA1c level | not serious | serious | not serious | not serious | not serious | 0.47 (0.18,0.75) | - | 409 | ⨁⨁⨁◯ Moderate |
| TG level | not serious | serious | not serious | not serious | serious | 0.07 (-0.16,0.31) | - | 139 | ⨁⨁◯◯ Low |
| CH level | not serious | serious | not serious | not serious | serious | 0.08 (-0.16,0.32) | - | 139 | ⨁⨁◯◯ Low |
| HDL level | not serious | serious | not serious | not serious | serious | -0.09 (-0.34,0.17) | - | 123 | ⨁⨁◯◯ Low |
| LDL level | not serious | serious | not serious | not serious | serious | 0.02 (-0.23,0.27) | - | 123 | ⨁⨁◯◯ Low |
| Clinical scales | not serious | serious | not serious | not serious | not serious | 1.50 (1.30,1.69) | - | 324 | ⨁⨁⨁◯ Moderate |
| HAMD Score | not serious | serious | not serious | not serious | not serious | 1.20 (0.97,1.43) | - | 204 | ⨁⨁⨁◯ Moderate |
| BDI score | not serious | serious | not serious | not serious | not serious | 1.54(1.10,1.98) | - | 58 | ⨁⨁⨁◯ Moderate |

Note: High quality: we are very confident that the true effect lies close to that of the estimate of the effect. Moderate quality: we are moderately confident in the effect estimate: the true effect is likely to be close to the estimate of the effect, but there is a possibility that it is substantially different. Low quality: our confidence in the effect estimate is limited: the true effect may be substantially different from the estimate of the effect. Very low quality: we have very little confidence in the effect estimate: the true effect is likely to be substantially different from the estimate of effect. SMD: standard deviation; CI: confidence interval.


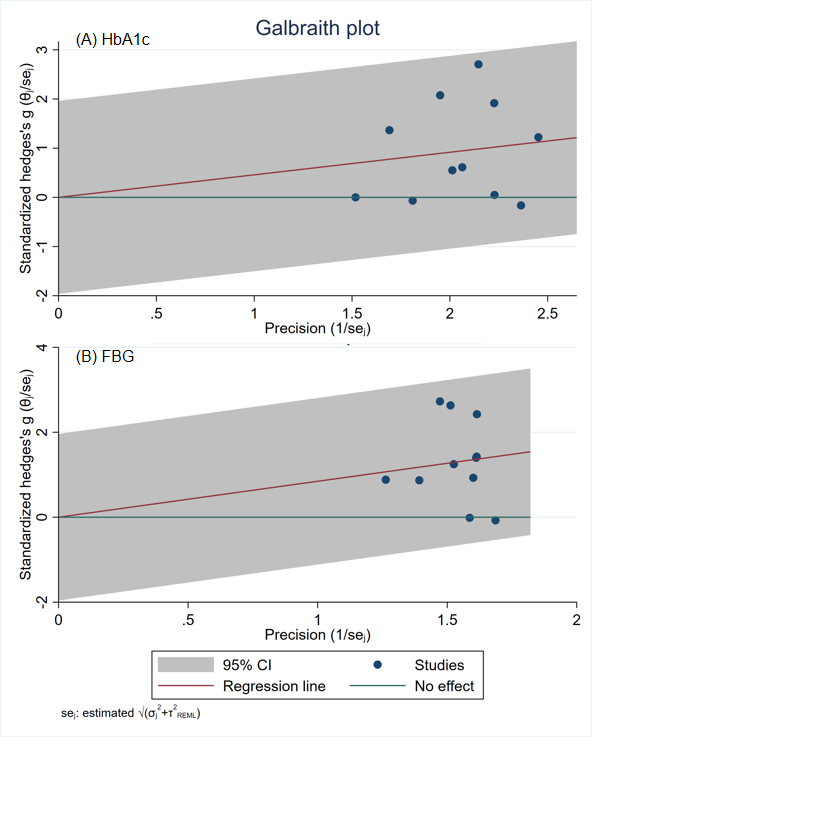


**Fig. S2 Galbraith plot for assessing heterogeneity (Glycemic Profiles)**

1. Galbraith plot of HbA1c groups. (B) Galbraith plot of FBG group.

**Table S3 Heterogeneity metrics**

| Meta regression | | FBG | | HbA1c | | TG | | CH | | HDL | | LDL | |
| --- | --- | --- | --- | --- | --- | --- | --- | --- | --- | --- | --- | --- | --- |
| Tau^2^ | | 0.19 | | 0.19 | | 0.07 | | 0 | | 0.11 | | 0.01 | |
| I^2^_res | | 62.83% | | 68.93% | | 100% | | 0 | | 100% | | 100% | |
| Model F/Wald chi2 | | 1.62 | | 0.38 | | 0.20 | | 1.07 | | 0.63 | | 0.07 | |
| Prob > F/chi2 | | 0.36 | | 0.77 | | 0.90 | | 0.59 | | 0.43 | | 0.79 | |
| Covariant | Study region | β= -0.90 | P=0.25 | β= -0.30 | P=0.36 | β= -0.29 | P= 0.67 | β= 0.20 | P= 0.73 | β= -0.52 | P= 0.43 | β= 0.13 | P= 0.79 |
|  | Age | β= -0.26 | P=0.62 | β= 0.13 | P=0.66 | β= 0.08 | P= 0.89 | β= -0.47 | P= 0.34 | - | - | - | - |
|  | Medication | β= -1.56 | P=0.23 | β= 0.08 | P=0.87 | - | - | - | - | - | - | - | - |
|  | Disorder | β= -1.50 | P=0.10 | - | - | - | - | - | - | - | - | - | - |

FBG, fasting blood glucose. HbA1c, glycosylated hemoglobin. TG, triglyceride. CH, cholesterol. HDL, high density lipoprotein. LDL, lower density lipoprotein.


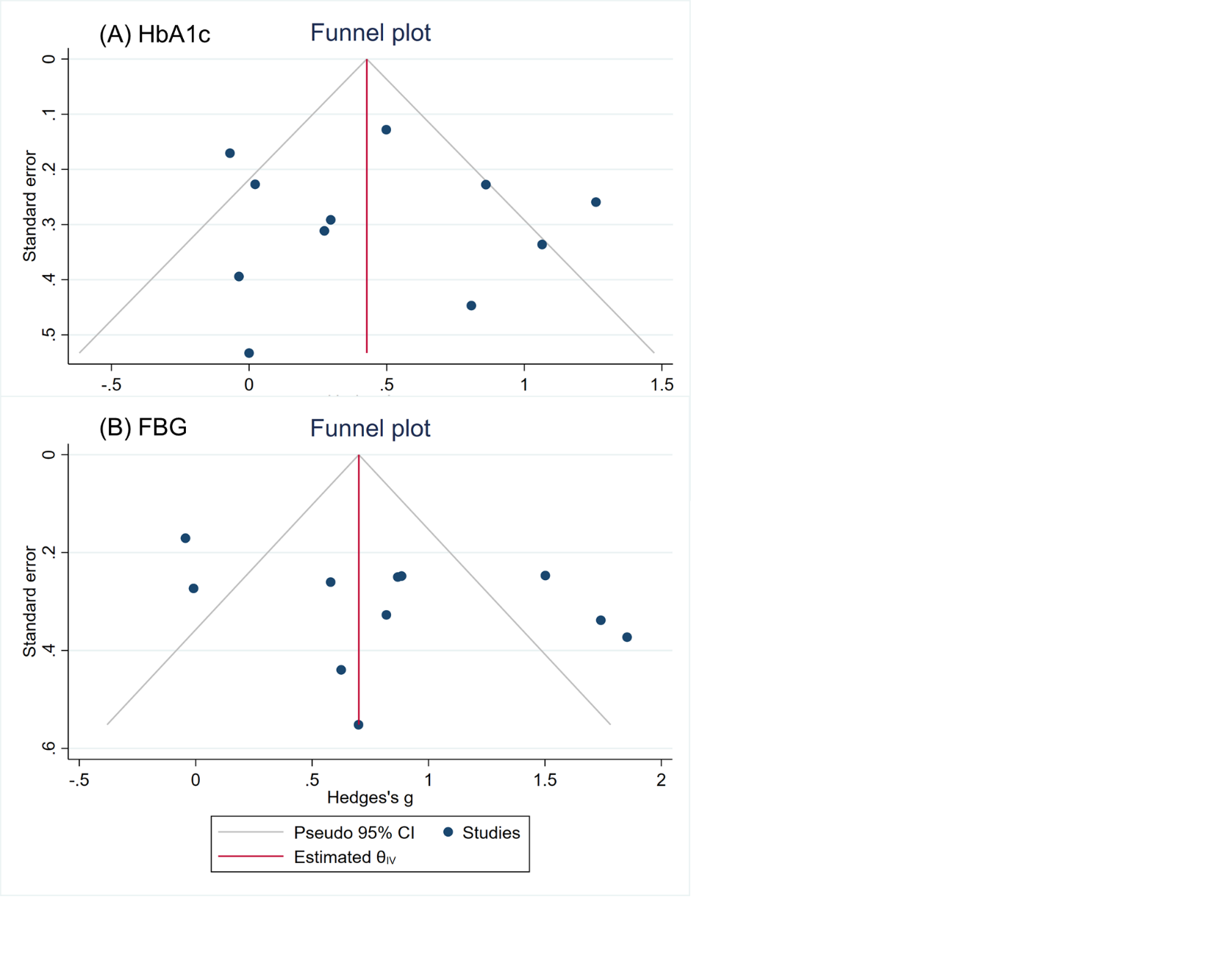


**Fig. S3 Funnel plot for assessing publication bias (Glycemic Profiles)**

1. Funnel plot of HbA1c groups. (B) Funnel plot of FBG group.


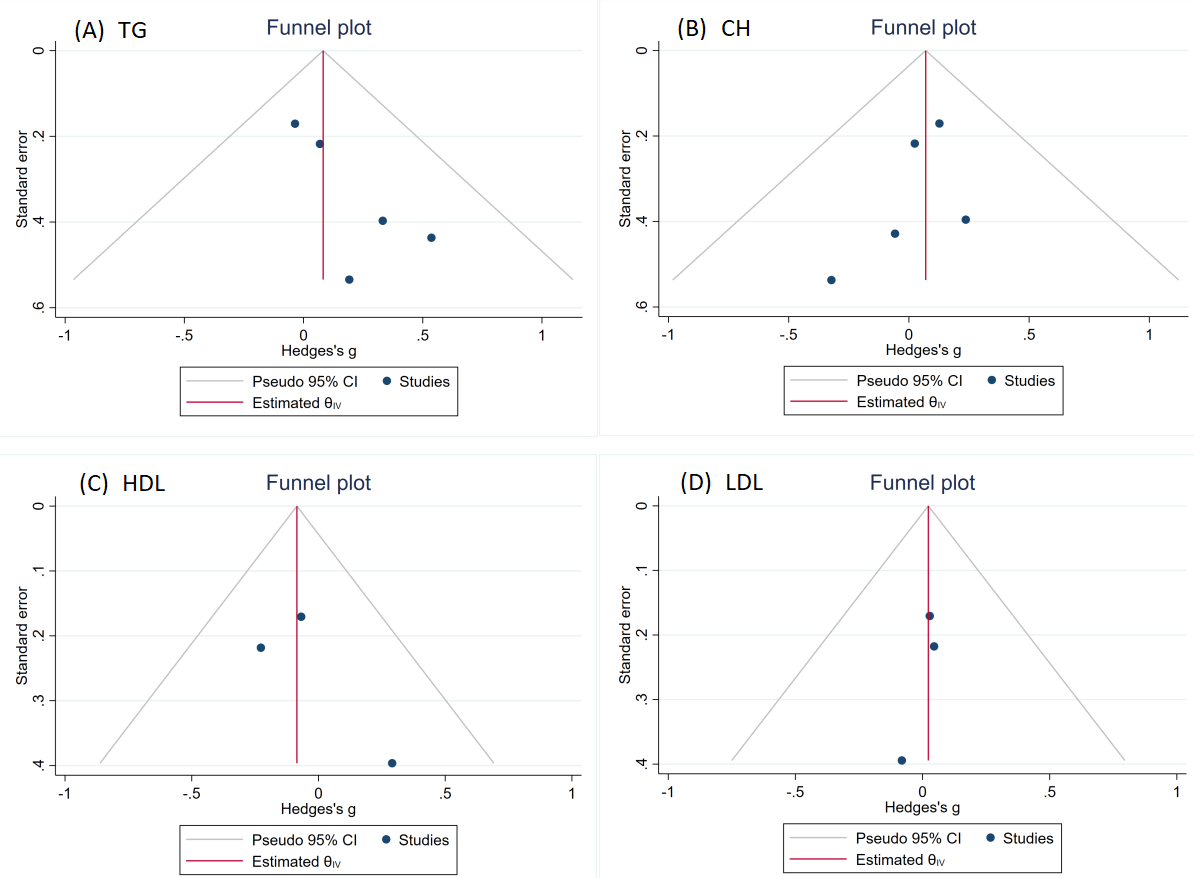


**Fig. S4 Funnel plot for assessing publication bias (Lipid Profiles)**

(A) Funnel plot of Triglyceride (TG) group. (B) Funnel plot of Cholesterol (CH) group. (C) Funnel plot of High-density lipoprotein (HDL) group. (D) Funnel plot of Low-density lipoprotein (LDL) group.

**Table S4 Publication bias assessment**

| Meta bias | | FBG | HbA1c | TG | CH | HDL | LDL |
| --- | --- | --- | --- | --- | --- | --- | --- |
| Egger test | Beta1 | 1.900 | 0.030 | -0.580 | 1.330 | 1.440 | -0.470 |
|  | SE of Beta1 | 2.204 | 1.445 | 1.098 | 1.092 | 1.904 | 1.913 |
|  | z | 0.860 | 0.020 | -0.530 | 1.220 | 0.760 | -0.240 |
|  | Prob > \|z\| | 0.388 | 0.982 | 0.595 | 0.222 | 0.449 | 0.808 |
| Begg’s test | Kendall’s score | 3.000 | 1.000 | -8.000 | 6.000 | 1.000 | -1.000 |
|  | SE of score | 11.180 | 12.845 | 4.082 | 4.082 | 1.915 | 1.915 |
|  | z | 0.180 | 0.000 | -2.200 | 1.220 | 0.000 | -1.04 |
|  | Prob > \|z\| | 0.858 | 1.000 | 0.086 | 0.221 | 1.000 | 1.000 |

FBG, fasting blood glucose. HbA1c, glycosylated hemoglobin. TG, triglyceride. CH, cholesterol. HDL, high density lipoprotein. LDL, lower density lipoprotein.

**
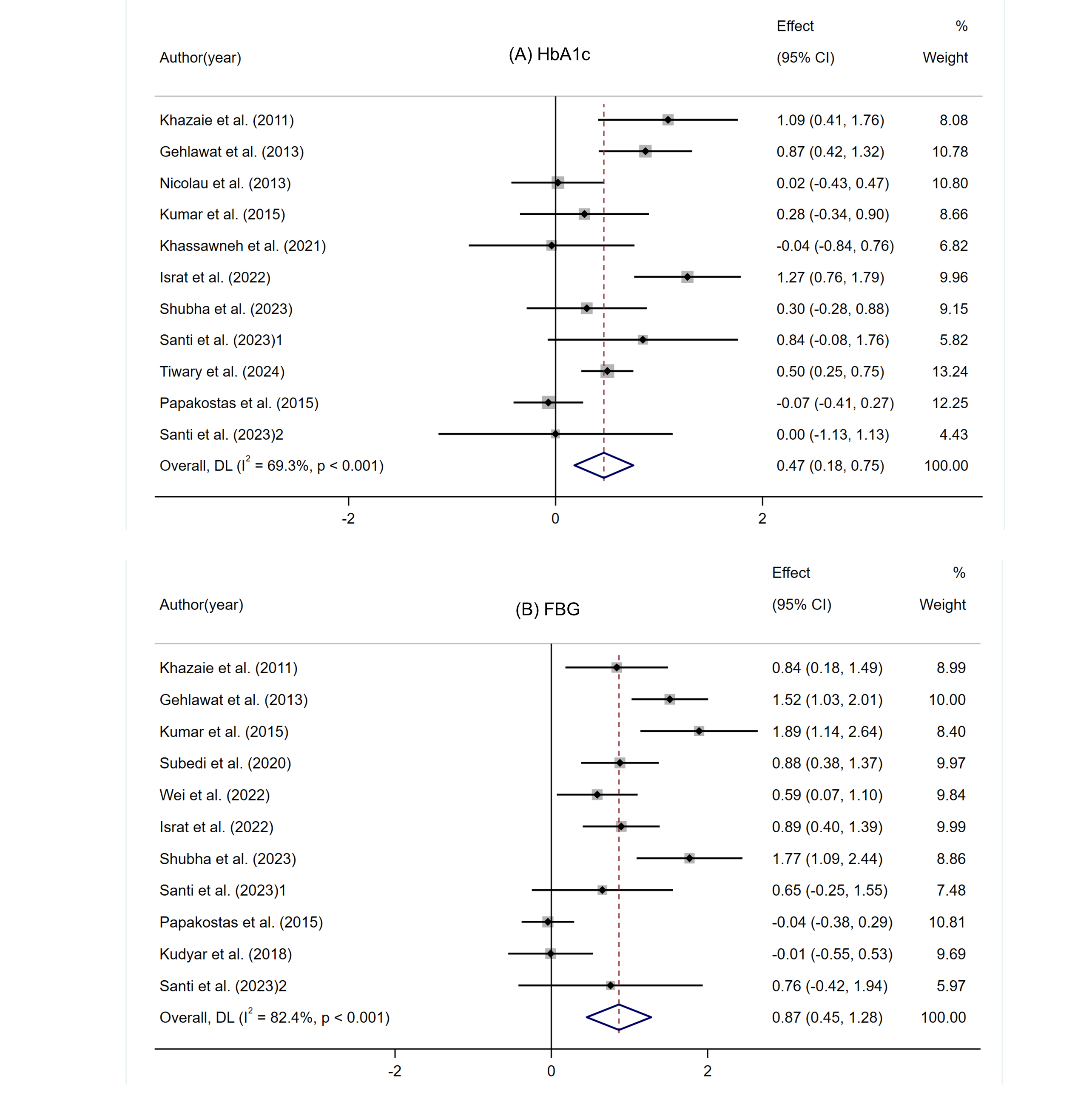
**

**Fig. S5 Changes in glucose metabolism before and after treatment**

(A) Comparison of HbA1c levels before and after treatment. (B) Comparison of FBG levels before and after treatment. Notes: Santi N. (2023)1 represents T2DM comorbid with MDD group, and Santi N. (2023)2 represents MDD only group.

**
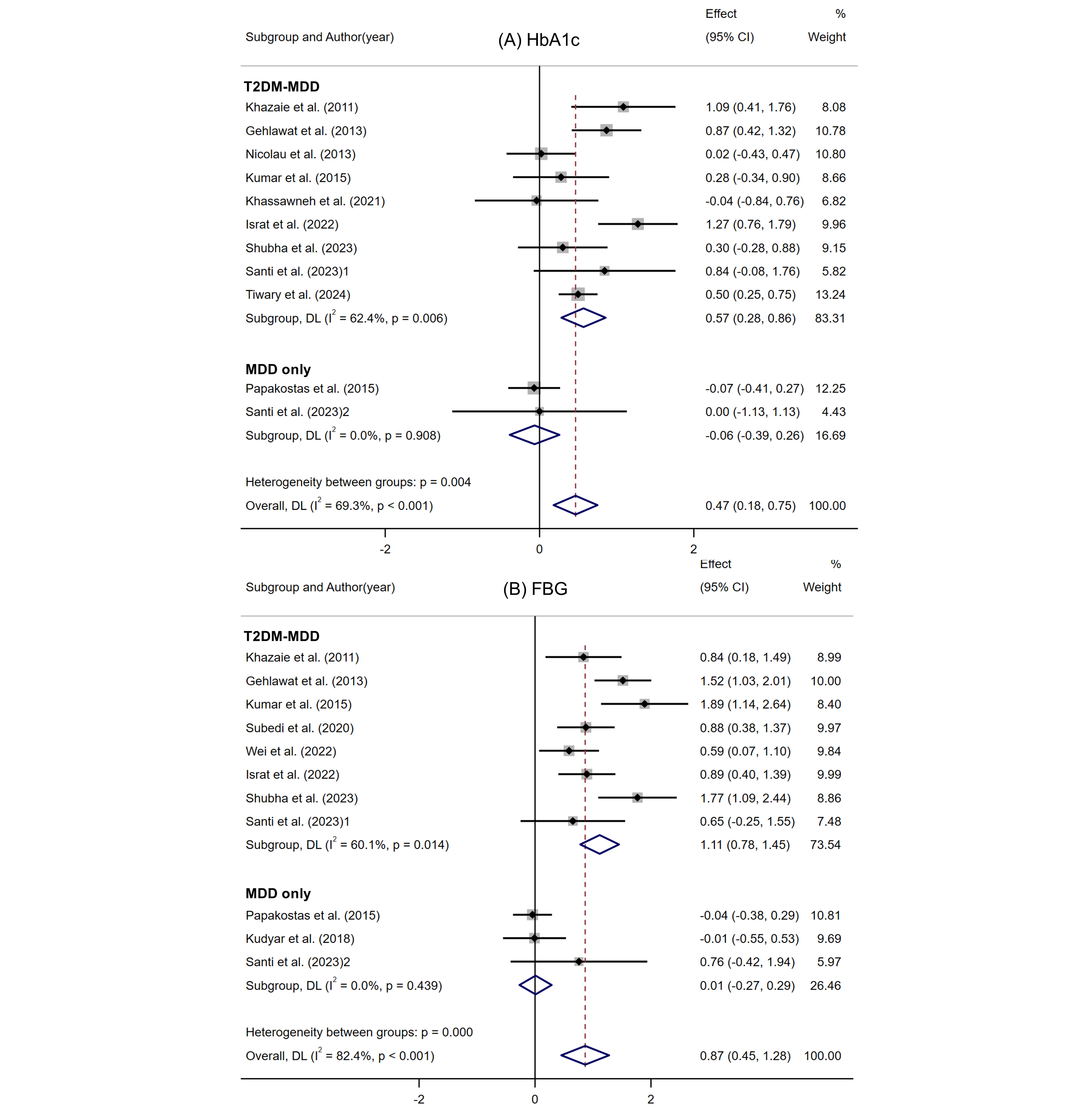
**

**Fig. S6 Subgroup analysis of T2DM+MDD group and MDD only group**

(A) HbA1c levels before and after treatment. (B) FBG levels before and after treatment. Notes: Santi N.(2023)1 represents T2DM comorbid with MDD group, and Santi N.(2023)2 represents MDD only group.

**
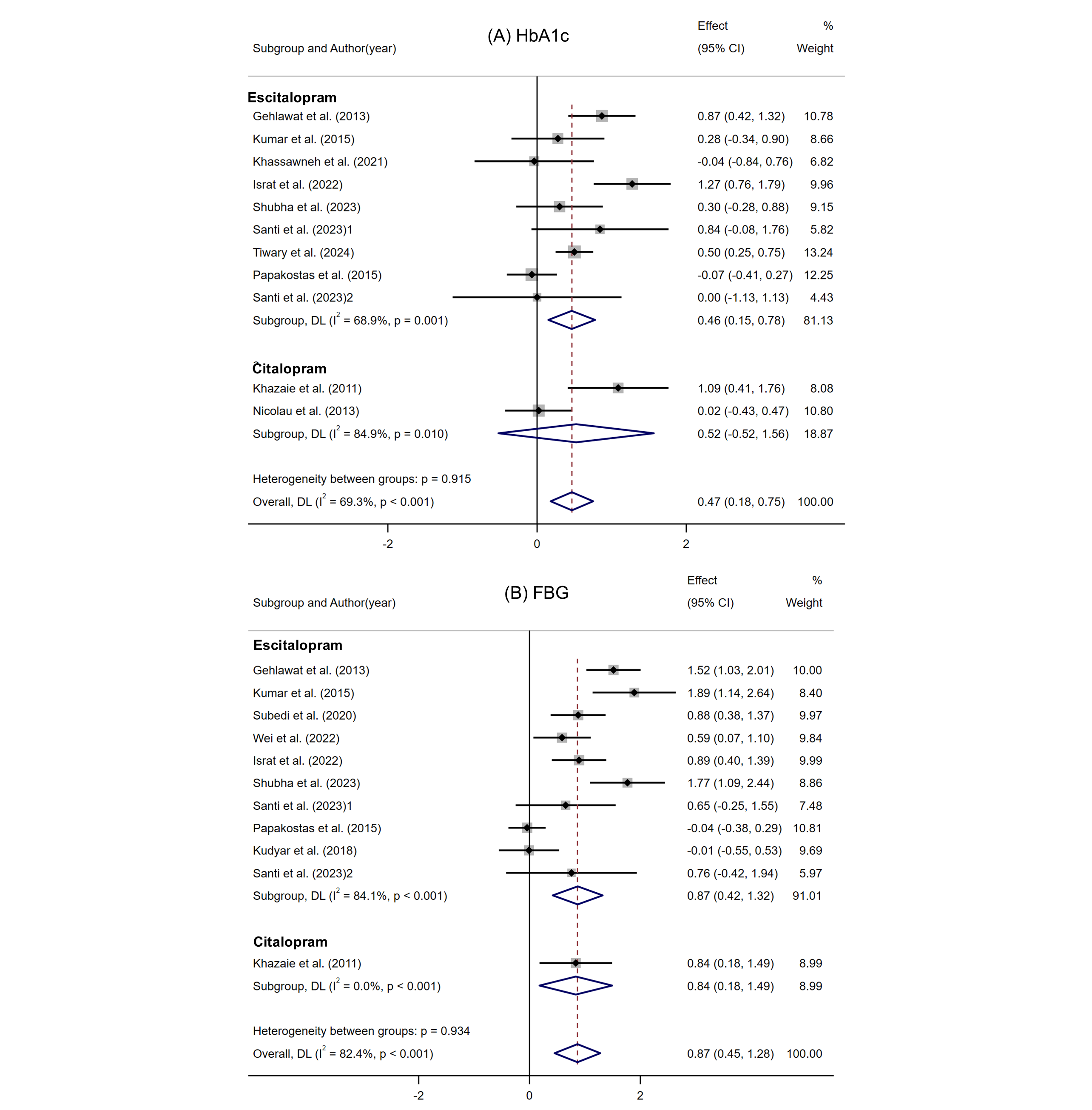
**

**Fig. S7 Subgroup analysis of Escitalopram group and Citalopram group**

(A) HbA1c levels before and after treatment. (B) FBG levels before and after treatment. Notes: Santi N.(2023)1 represents T2DM comorbid with MDD group, and Santi N.(2023)2 represents MDD only group.

**Table S5 Changes in lipid measures of the included studies**

| **Study** | **Intervention** | **Baseline** | | | | **Post-treatment** | | | |
| --- | --- | --- | --- | --- | --- | --- | --- | --- | --- |
|  |  | **TG (mg/dl)** | **CH (mg/dl)** | **HDL(mg/dl)** | **LDL (mg/dl)** | **TG (mg/dl)** | **CH (mg/dl)** | **HDL(mg/dl)** | **LDL (mg/dl)** |
| Gehlawat et al. (2013) | E:10-20mg/d | 181.28±84.70 | 195.63±51.22 | 42.53±7.58 | 109.98±33.30 | 179.20±83.55 | 192.10±50.73 | 44.75±7.58 | 108.48±32.23 |
| Khassawneh et al. (2021) | E:5-10mg/d | 180.53±116.88 | 182.91±31.71 | 49.16±29.80 | 117.96±26.71 | 155.84±81.46 | 168.21±51.43 | 42.19±13.55 | 120.52±33.69 |
| Santi et al.(2023)^1^ | E:10-20mg/d | 120.50±33.37 | 160.00±20.53 | NR | NR | 122.50±32.96 | 148.50±20.54 | NR | NR |
| Papakostas et al.(2015) | E:10-30mg/d | 186.90±138.50 | 191.30±42.60 | 52.40±26.60 | 110.10±34.40 | 166.60±177.0 | 193.00±51.50 | 54.30±28.90 | 109.00±42.40 |
| Santi et al.(2023)^2^ | E:10-20mg/d | 124.50±20.24 | 152.00±9.26 | NR | NR | 92.50±3.48 | 149.00±18.19 | NR | NR |

E, Escitalopram. TG, Triglyceride. CH, total cholesterol. HDL, high density lipoprotein. LDL, lower density lipoprotein. NR, not reported. Data represent as the mean ± Standard Deviation. Santi et al.(2023) 1 represents T2DM comorbid with MDD group, and Santi et al.(2023) 2 represents MDD only group.


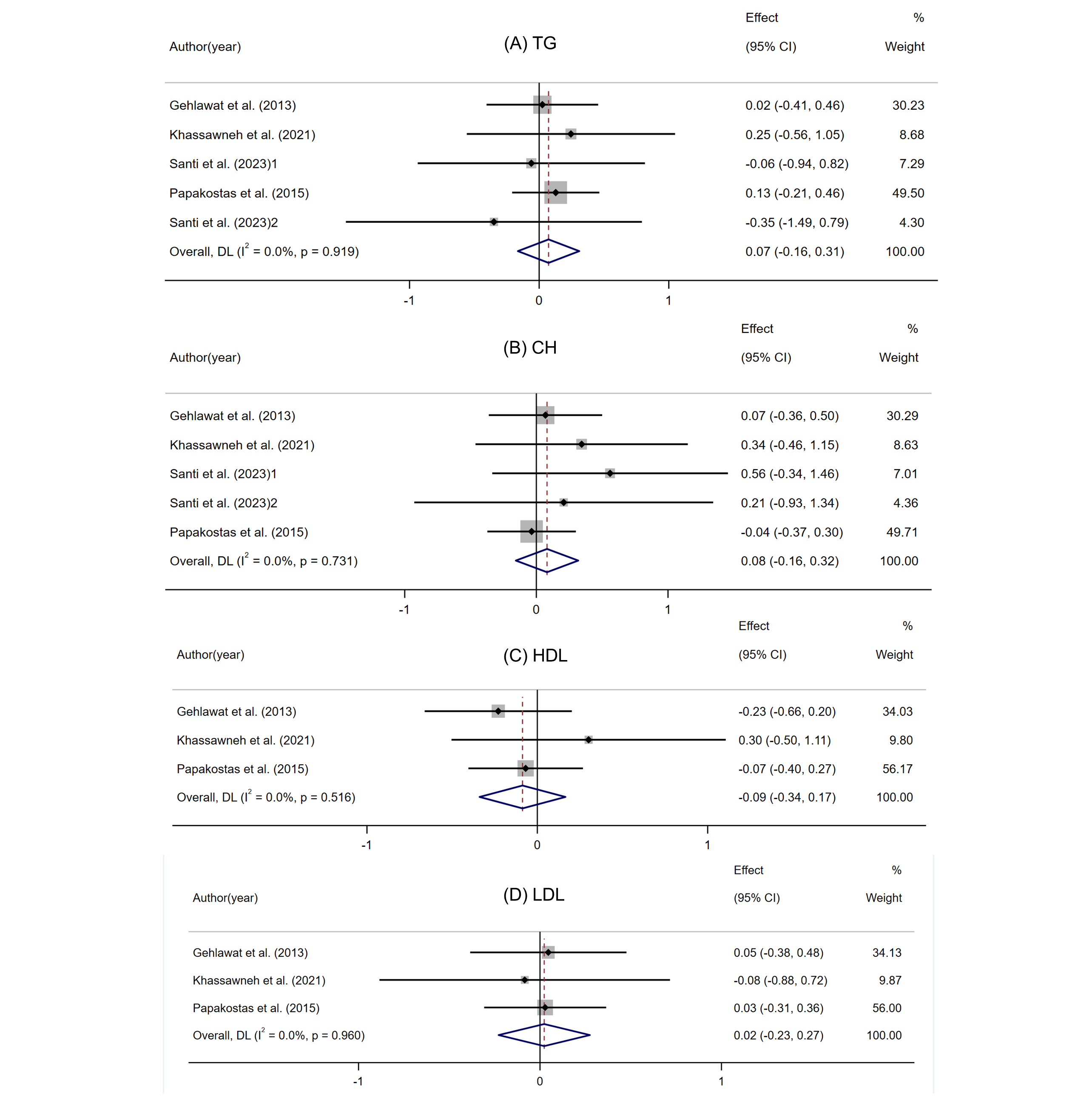


**Fig. S8 Changes in lipid metabolism before and after treatment with citalopram or escitalopram**

(A)Triglyceride (TG) levels. (B) Cholesterol (CH) levels. (C) High-density lipoprotein (HDL) levels. (D) Low-density lipoprotein (LDL) levels. Note: Santi N.(2023)1 represents T2DM comorbid with MDD group, and Santi N.(2023)2 represents MDD only group.


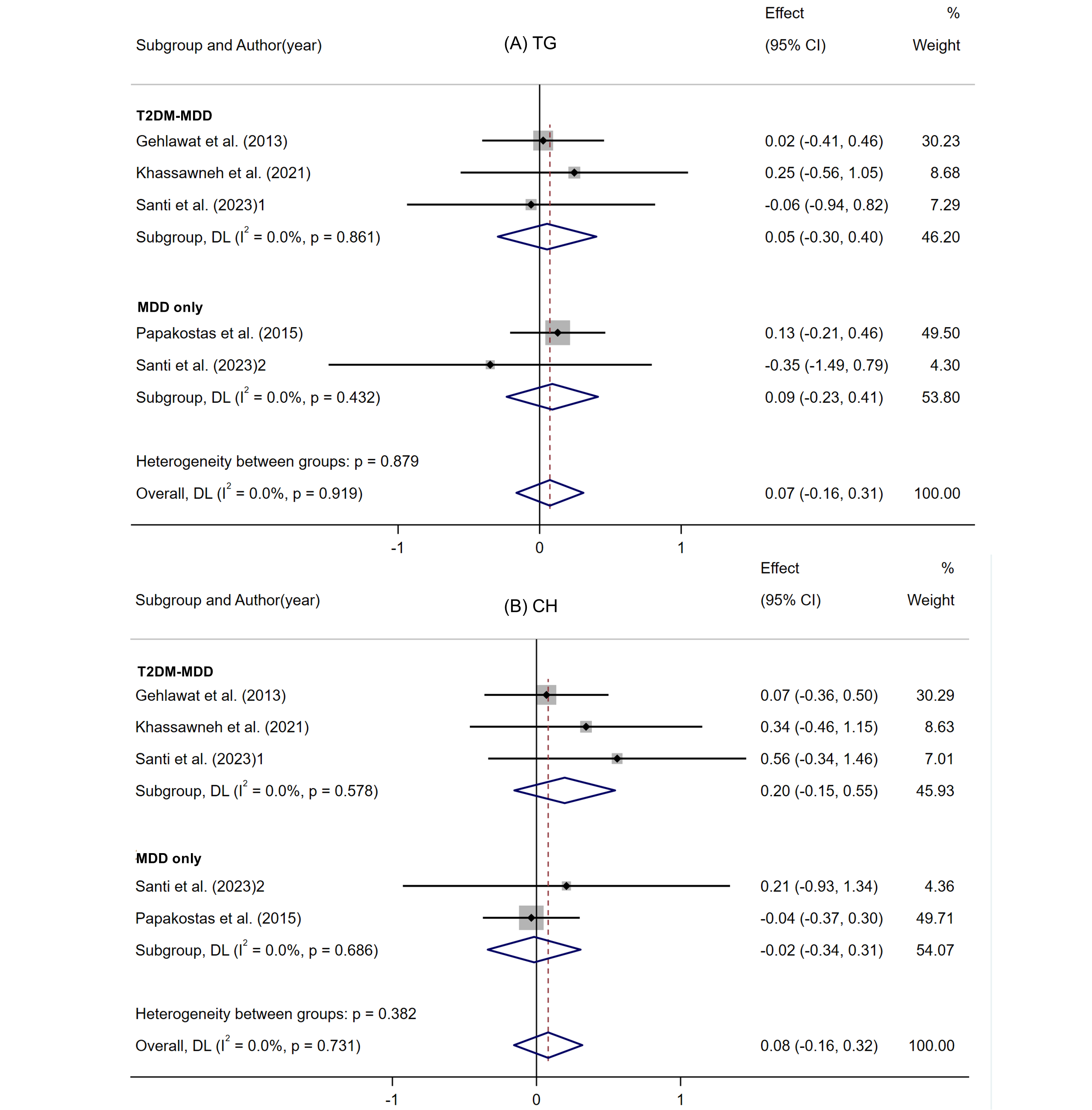


**Fig. S9 Subgroup analysis of lipid metabolism changes in T2DM+MDD and MDD only groups**

1. Triglyceride (TG) levels. (B) Cholesterol (CH) levels. Note: Santi N.(2023)1 represents T2DM comorbid with MDD group, and Santi N.(2023)2 represents MDD only group.


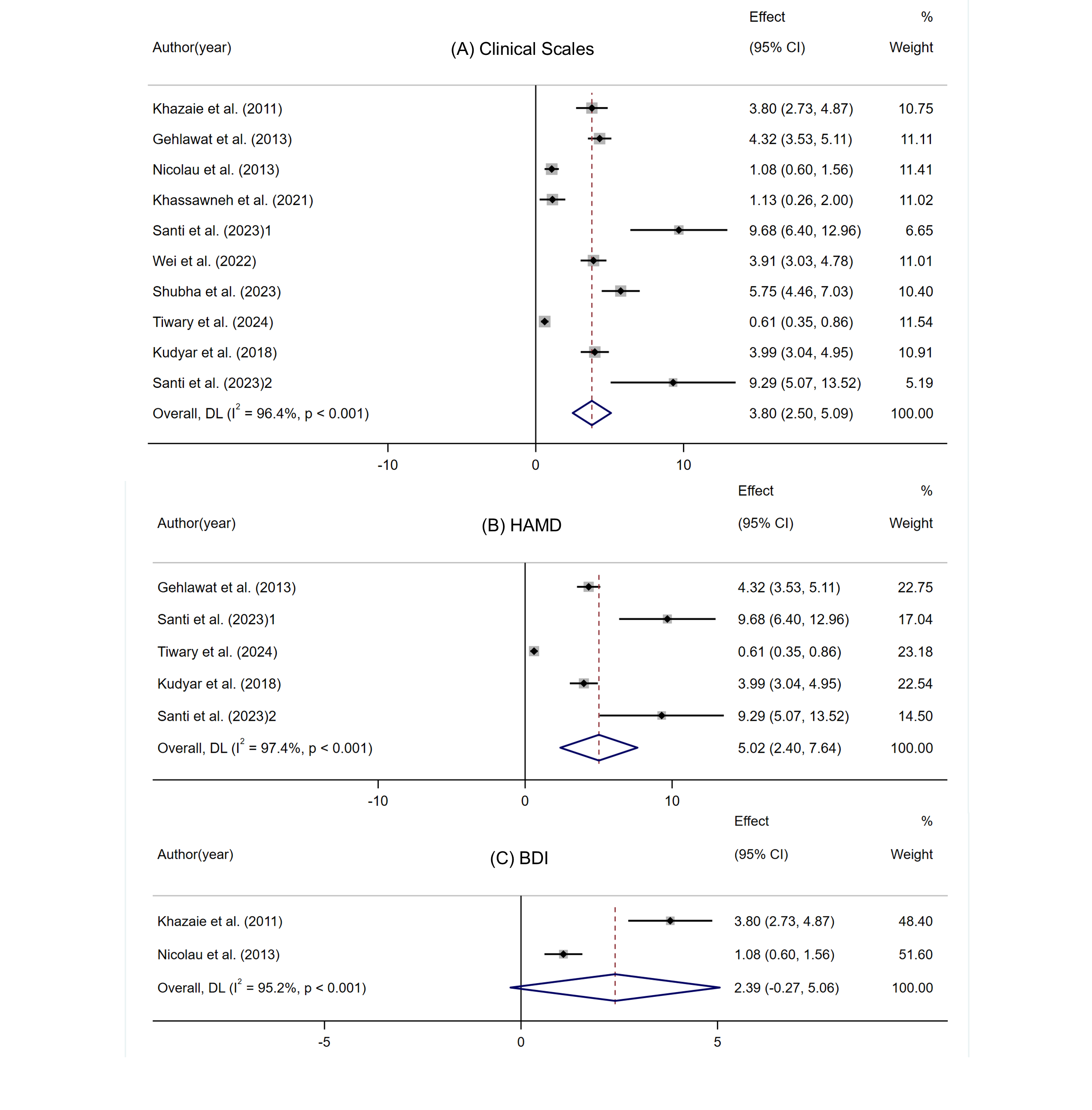


**Fig. S10 Changes in depression and anxiety assessment scales before and after treatment with citalopram or escitalopram.**

1. Comparison of overall clinical scores. (B) Comparison of Hamilton Depression Rating Scale (HAMD) scores. (C) Comparison of Beck Depression Inventory (BDI) scores. Note: Santi N.(2023)1 represents T2DM comorbid with MDD group, and Santi N.(2023)2 represents MDD only group.
