# Supplementary figures and images for: Effects of the selective serotonin reuptake inhibitors citalopram and escitalopram on glucolipid metabolism: a systematic review
Source: Front Endocrinol (Lausanne). 2025 Jun 17;16:1578326. doi: 10.3389/fendo.2025.1578326 (PMC12209303; doi:10.3389/fendo.2025.1578326)

## Slide 1
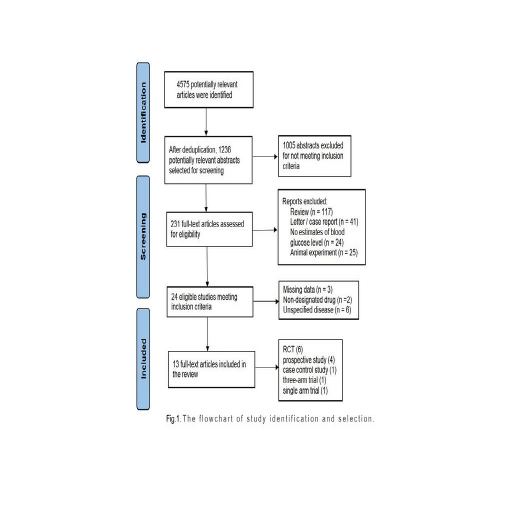

## Slide 2
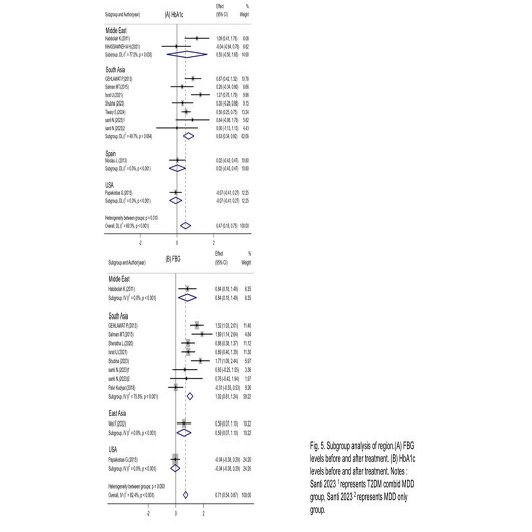

## Slide 3
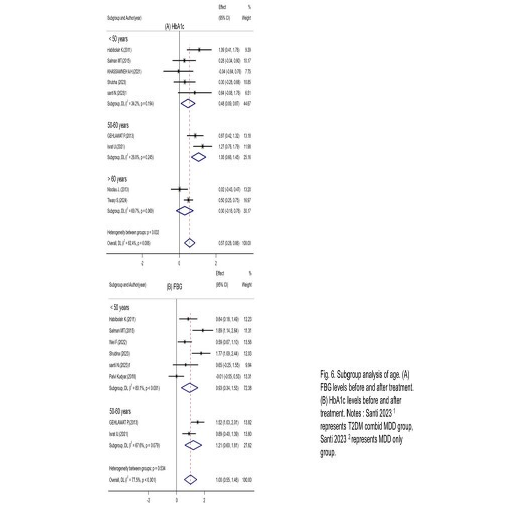

Supplement: Supplementary file 3 [file Presentation1.pptx]
